# High Prevalence of HIV, HCV and Tuberculosis and Associated Risk Behaviours among New Entrants of Methadone Maintenance Treatment Clinics in Guangdong Province, China

**DOI:** 10.1371/journal.pone.0076931

**Published:** 2013-10-08

**Authors:** Lei Zhang, Di Zhang, Wen Chen, Xia Zou, Li Ling

**Affiliations:** 1 Sun Yat-sen Center for Migrant Health Policy, Sun Yat-sen University, Guangzhou, P. R. China; 2 School of Public Health, Sun Yat-sen University, Guangzhou, P. R. China; 3 The Kirby Institute, University of New South Wales, Sydney, Australia; 4 Office of Medical Science, Sun Yat-sen University, Guangzhou, P. R. China; Temple University School of Medicine, United States of America

## Abstract

**Background:**

Methadone maintenance treatment (MMT) has been available in Guangdong province, China since 2006. This study aims to estimate the prevalence levels of HIV, Hepatitis C (HCV), Tuberculosis (TB) and their co-infections and associated demographic and risk behaviours among MMT entrants.

**Method:**

A total of 2296 drug users at the time of their MMT enrolment were recruited from four clinics during 2006-2011. Participants’ demographic characteristics, infection status and self-reported high-risk drug-use and sexual behaviours were surveyed. Log-linear contingency analysis was employed to investigate the demographic and behavioural differences between gender and drug-user type, while multivariate regression analysis was used to identify the associated factors of HIV, HCV and TB infections.

**Results:**

Female drug users demonstrate significantly higher frequency of daily drug consumption (Log-linear contingency analysis, G^2^=10.86, *p*=0.013) and higher proportion of having had sex in the past three months (G^2^=30.22, *p*<0.001) than their male counterparts. Among injecting drug users, females also inject (χ^2^=16.15, *p*=0.001) and share syringes (χ^2^=13.24, *p*=0.004) more frequently than males. Prevalence of HIV, HCV and TB among MMT entrants are 6.3%, 78.7% and 4.4% respectively. Co-infections of HIV/HCV, HIV/TB, HCV/TB and HIV/HCV/TB reportedly infect 5.6%, 0.5%, 3.8% and 0.3% of study participants. Infection risks of HIV, HCV and TB are consistently associated with increasing length of drug use, injecting drugs, financial dependence and reduced sexual activities.

**Conclusion:**

Injecting drug use is the major contributing factor in prevalence levels of HIV, HCV and TB among MMT entrants. Female drug users are more disadvantaged in their social status and risk-taking in their drug use behaviours than males.

## Introduction

The wide spread of Human Immunodeficiency Virus (HIV), hepatitis C virus (HCV) and tuberculosis (TB) infections are three major public health issues, both globally and in China. Drug users, particularly injecting drug users (IDUs), are one of the most at-risk populations for these infections [1-3]. China has the largest population of drug users in the world, which currently numbers 3.4 million [4,5], among whom two-third are IDUs [6]. The most recent literature reported the national prevalence of HIV and HCV among drug users to be 4.1% and 50.4% respectively [6-8]. Approximately 1.9% of Chinese drug users are co-infected with HIV/HCV [9], and TB accounts for more than a third of the opportunistic infections among drug users living with HIV in China [10]. High frequency of injections and sharing of injecting equipment are the primary risk behaviours contributing to HIV and HCV transmission, whereas deleterious effects of drug use on the immune system, combined tobacco and alcohol consumption, homelessness and incarceration are major risk factors for TB infection among drug users [6,11-16].

Methadone maintenance treatment (MMT) is widely known to be an effective harm reduction strategy to reduce high-risk drug behaviours and HIV transmission among drug users in China [17,18]. China initiated its pilot MMT program in eight clinics in 2004 [19]. Since then, the MMT program has been substantially scaled-up nationwide. By 2011, a total of 716 clinics had been established across the country, serving 332,996 drug users. A large number of new enrolments have been reported each year. The Chinese MMT program has become one of the largest single drug treatment and care systems in the world. Most MMT entrants are registered drug users under the administration of the Ministry of Public Security. These entrants represent a subgroup of high-risk drug users, as they often have a history of incarceration and fail multiple detoxification attempts [20]. A large number of studies have been undertaken to identify the demographic characteristics, HIV and HCV disease burden among MMT entrants to better inform MMT implementation strategies and practices in China [9,21-23]. Although these studies provide important primary descriptions about participants of MMT programs, several knowledge gaps remain. First, the disease burden of TB among MMT entrants has not been reported. Second, although male drug users account for the majority of MMT participants, female drug users disproportionally bear an extra risk of HIV infection as a high proportion of them also participate in commercial sex work [24]; however, the high-risk drug use and sexual behavioural patterns of female drug users are under-reported. Third, the associated behavioural factors of HIV, HCV and TB infections have not been investigated specifically for MMT entrants.

Based on the enrolment information of 2296 MMT clients in four selected clinics in Guangdong during 2006-2011, this study aims to (1) identify the differences in demographics characteristics and risk behaviours caused by gender and injecting behaviours among MMT entrants; and (2) investigate the underlying associated factors of HIV, HCV and TB infections to provide evidence to inform health policies and better practices in MMT clinics in China.

## Methods

### Study sites

Guangdong province in Southern China is one of the six Chinese provinces most severely affected by the HIV epidemic, and drug users are at high risk [25,26]. By 2011, 58 MMT sites had been established and serve a total 25,923 registered drug users. This study was conducted at four MMT clinics located in the cities of Guangzhou, Jiangmen, Taishan and Shenzhen in Guangdong province. These four clinics were selected because they were the first clinics established (during October 2006 and January 2007) in Guangdong province. They retained the most complete record of participants and are the most representative of MMT program in Guangdong.

### Participants

Entrants were regarded as injecting drug users if they satisfied one of the following conditions: 1) current type of drug used was ‘injecting’ or ‘mixed’; 2) had self-reported a history of injecting drugs or sharing syringes in the previous 30 days; 3) injecting or syringe sharing frequency in the previous 30 days was more than once. Other participants were classified as non-IDUs.

This study included all entrants from all four MMT clinics during 2006-2011 who met the eligibility criteria. An MMT entrant is eligible for inclusion if he/she was: 1) ICD-10 diagnosed with current opioid dependence; 2) 18 years or above; 3) a local resident who settled in the catchment areas of the clinics; 4) provided written informed consent for the use of their clinical data in MMT databases; 5) enrolled in MMT for the first time; and 6) had a clearly defined drug-use profile. Based on these criteria, 2296 MMT entrants were included.

### Data collection

Information on all new entrants, including demographic characteristics, self-reported drug use and sexual behaviours, was collected by a comprehensive and self-administered questionnaire. The questionnaire was based on the standardised MMT survey in China [27] at the time of enrolment. All information was confidentially recorded by clinic staff and de-identified for privacy protection. According to the national guidelines for MMT [27], all MMT entrants were required to test for HIV, HCV and TB at enrolment. Blood samples were collected at MMT clinics for primary screening. HIV and HCV infections were screened with enzyme-linked immunosorbent assay (ELISA) and immunocolloidal golden method, with positive results confirmed by Western blot and ELISA respectively in designated laboratories of the local Center for Disease Prevention and Control (CDC). Clinical diagnosis and X-ray chest screening for TB infections was also conducted according to the national TB diagnostic procedure [28]. Suspected cases were referred to hospitals for further confirmation tests and appropriate treatment. However, hospitals and clients were not obliged to report the results of confirmation tests back to the MMT clinics.

### Ethical considerations

All participants provided written consent for the use of their personal data in the MMT clinics’ database for the purpose of this study. The study was approved by the Ethics Committee of School of Public Health, Sun Yat-sen University, China (Proposal number 71173245,30972552).

### Statistical analysis

A log-linear contingency analysis approach was employed to compare the demographic characteristics, high-risk drug use and sexual behaviours between gender (male/female) and drug-user type (IDUs/non-IDUs). This approach takes into consideration the possible intrinsic interaction between the gender and drug-user type and provides more accurate results than two separate one-way contingency analyses [29,30]. Linear-regression was conducted to assess the temporal trends of the prevalence levels. All temporal variations were insignificant and hence data in all years were pooled for analysis. Univariate logistic regression models were employed to examine the associations between a comprehensive set of demographic and risk factors and each of HIV, HCV and TB prevalence levels (Table S1). Resulting factors with *p*<0.10 in the univariate analysis were then included as candidates for backward multivariate logistic regression analysis. Odd ratios (ORs), together with 95% confidence intervals, were presented. All analyses were conducted using PASW software version 18.0.

## Results

### Demographic Characteristics

A total of 2296 eligible MMT entrants were included in this study. Among these, 87.4% was male and 79.7% was self-identified as IDUs. In general, most of these participants were in their late 30s (38.9±6.6 years), had ever been married or divorced (53.3%), were poorly educated (77.5%), unemployed (61.2%) and financially reliant on their families (52.5%, Table 1). Demographic characteristics were substantially different between genders and type of drug users. In particular, male drug users were usually two to three years older than their female counterparts (2-way contingency analysis, *G* ^*2*^ *=24.32, p<0.001*), but more likely to be single (47.2% versus 42.9%, *G* ^*2*^ *=10.10, p=0.018*). They were significantly less well-educated (senior high or above, 21.5% male versus 29.4% female, *G* ^*2*^ *=8.64, p=0.003*) but more likely to have a stable salary as source of income (45.5% male versus 36.7% female, *G* ^*2*^ *=8.48, p=0.037*) at time of enrolment. Compared with non-IDUs, IDUs were significantly more likely to be single (50.0% versus 33.5%, *G* ^*2*^ *=46.98, p<0.001*), less well-educated (20.2% versus 31.4%, *G* ^*2*^ *=25.14, p<0.001*), and relied on their own income for living (42.1% versus 53.5%, *G* ^*2*^ *=19.88, p<0.001*). Given these differences in demographic characteristics, female IDUs appeared to be the most disadvantaged subgroup with the lowest socio-economic status.

**Table 1:** Demographic information, risk drug-use and sexual behaviours among 2296 MMT entrants during 2006-2011 in Guangdong province, China.

	**Total**	**IDU**		**Non-IDU**		**Log-linear Contingency Analysis**	
	**(n=2296)**	**Male (n=1629, %)**	**Female (n=202, %)**	**Male (n=378, %)**	**Female (n=87, %)**	**Gender *p* (G^2^)**	**Drug-user type *p* (G^2^)**
***DemographicCharacteristics***							
**Age**							
*21-30*	207 (9.0)	137 (8.4)	28 (13.9)	31 (8.2)	11 (12.6)	<0.001	0.850
*31-40*	1232 (53.7)	862 (52.9)	124 (61.4)	193 (51.1)	53 (60.9)	(24.32)	(0.80)
*41-50*	756 (32.9)	559 (34.3)	44 (21.8)	132 (34.9)	21 (24.1)		
*≥51*	101 (4.4)	71 (4.4)	6 (3.0)	22 (5.8)	2 (2.3)		
*Average* (Mean±SD)	38.9±6.6	39.1±6.5	36.8±6.3	39.7±7.0	37.0±6.4		
**Marital status**								
*Single*	1072 (46.7)	822 (50.5)	94 (46.5)	126 (33.3)	30 (34.5)	0.018	<0.001
*Married*	979 (42.6)	643 (39.5)	75 (37.1)	216 (57.1)	45 (51.7)	(10.10)	(46.98)
*Divorced*	238 (10.4)	161 (9.9)	31 (15.3)	35 (9.3)	11 (12.6)		
*Widowed*	7 (0.3)	3 (0.2)	2 (1.0)	1 (0.3)	1 (1.1)		
**Education level**							
*Junior high or below*	1780 (77.5)	1307 (80.2)	154 (76.2)	269 (71.2)		50 (57.5)	0.003	<0.001
*Senior high or above*	516 (22.5)	322 (19.8)	48 (23.8)	109 (28.8)	37 (42.5)	(8.64)	(25.14)
**Major source of income**							
*Salary*	1019 (44.4)	698 (42.9)	72 (35.7)	215 (56.9)	34 (39.0)	0.037	<0.001
*Family/friends*	1205 (52.5)	875 (53.7)	124 (61.4)	156 (41.3)	50 (57.5)	(8.48)	(19.88)
*Social Welfare*	58 (2.5)	44 (2.7)	3 (1.5)	6 (1.6)	3 (3.4)		
*Missing*	16 (0.7)	12 (0.7)	3 (1.5)	1 (0.3)	0 (0.0)		
***Drug-usebehavioursatbaseline***							
**Duration of drug usage**							
*≤10 year*	841 (36.6)	515 (31.6)	89 (44.1)	184 (48.7)	53 (60.9)	<0.001	<0.001
*11-20 year*	1338 (58.3)	1028 (63.1)	102 (50.5)	175 (46.3)	33 (37.9)	(22.82)	(50.68)
*21-30 years*	108 (4.7)	80 (4.9)	9 (4.5)	18 (4.8)		1 (1.1)		
>30 years	9 (0.4)	6 (0.4)	2 (1.0)	1 (0.3)	0 (0.0)		
*Average* (Mean±SD)	13.0±5.7	13.0±5.2	11.7±5.9	10.9±6.0	9.3±5.3		
**Current type of drug used**							
*Heroin*	2185 (95.2)	1548 (95.0)	193 (95.5)	360 (95.2)	84 (96.6)	0.252	0.044
*others*	34 (1.5)	29 (1.8)	2 (1.0)	3 (0.8)	0 (0.0)	(2.76)	(6.26)
*Mixed Heroin & others*	40 (1.7)	33 (2.0)	3 (1.5)	4 (1.1)	0 (0.0)		
*Missing*	37 (1.6)	19 (1.2)	4 (2.0)	11 (2.9)	3 (3.4)		
**Frequency of daily drug usage in the last 30 days**							
*≤1 times / day*	170 (7.4)	100 (6.1)	10 (5.0)	51 (13.5)	9 (10.3)	0.013	<0.001
*1-2 times / day*	554 (24.1)	391 (24.0)	42 (20.8)	103 (27.2)	18 (20.7)	(10.86)	(29.18)
*3-5 times / day*	1370 (59.7)	1005 (61.7)	126 (62.4)	194 (51.3)	45 (51.7)		
*>5 times/day*	168 (7.4)	117 (7.2)	22 (10.9)	16 (4.2)	13 (14.9)		
*Missing*	34 (1.5)	16 (1.0)	2 (1.0)	14 (3.7)	2 (2.3)		
*Average* (Mean±SD)	3.5±1.9	3.3±1.7	3.7±2.1	2.9±1.8	3.7±2.5		
**Injecting drugs in the last 30 days**							
*Yes*	1724 (75.1)	1528 (93.8)	196 (97.0)	0 (0.0)	0 (0.0)	0.003	<0.001
*No*	568 (24.7)	100 (6.1)	5 (2.5)	376 (99.5)	87 (100.0)	(8.62)	(1762.76)
*Missing*	4 (0.2)	1 (0.1)	1 (0.5)	2 (0.5)	0 (0.0)		
***Sexualbehavioursatbaseline***							
**Have sex in the past 3 months**							
*Yes*	1523 (66.3)	1044 (64.1)	145 (71.8)	272 (72)	62 (71.3)	<0.001	0.004
*No*	753 (32.8)	571 (35.1)	55 (27.2)	104 (27.5)	23 (26.4)	(30.22)	(8.20)
*Missing*	20 (0.9)	14 (0.9)	2 (1.0)	2 (0.5)	2 (2.3)		
**Number of sexual partners in the past 3 moths**							
*0*	236 (10.3)	181 (11.1)	27 (13.4)	25 (6.6)	3 (3.4)	0.493	<0.001
*1*	1135 (49.4)	756 (46.4)	98 (48.5)	225 (59.5)	56 (64.4)	(3.40)	(25.80)
*2-5*	136 (5.9)	97 (6.0)	16 (7.9)	20 (5.3)	3 (3.4)		
*>6*	12 (0.5)	7 (0.4)	4 (2.0)	1 (0.3)	0 (0.0)		
*Missing*	777 (33.8)	588 (36.1)	57 (28.2)	107 (28.3)	25 (28.7)		
*Average*	1.2±3.6	1.1±3.2	1.4±2.6	1.4±5.4	1.0±0.4		
**Condom Usage in the last sex act**							
*Yes*	503 (21.9)	329 (20.2)	47 (23.3)	106 (28.0)	21 (24.1)	1.000	0.030
*No*	1018 (44.3)	713 (43.8)	99 (49.0)	166 (43.9)	40 (46.0)	(0.00)	(4.74)
*Missing*	775 (33.8)	587 (36.0)	56 (27.7)	106 (28.1)	26 (29.9)		

### High-risk behaviours

Drug usage duration was 13.0±5.7 years and heroine was the main substance among MMT entrants (95.2%, Table 1). In the past 30 days, the average frequency of daily drug use was 3.5±1.9 and 75.1% of drug users had injected. Among the 1724 injectors, the average injecting frequency is 86.6±55.5 in the past 30 days, and 14% indicated sharing syringes with others. Among those who shared, they shared on 35.4±60.4 occasions with 1.4±2.6 needle partners in the same period (Table 2). Notably, female participants were more risk-taking than males, evidenced by the significantly higher frequency of drug use (Table 1, ≥3 time/day, 72.7% female versus 67.9% male, *G* ^*2*^ *=10.86, p=0.013*) and a higher proportion of injection in the past 30 days (97.0% versus 93.8%, *G* ^*2*^ *=8.62, p=0.003*). Further, females IDUs injected significantly more frequently than males on a monthly base (Table 2, ≥100 episodes/month, 42.3% versus 29.3%, *χ* ^*2*^ *=16.15, p=0.001*) and also shared on more occasions (≥100 episodes/month, 25.9% versus 7.5%, *χ* ^*2*^ *=13.24, p=0.004*). Consistent with younger age, female drug users have a significantly shorter drug using history than male IDUs (1.3-1.6 years, *G* ^*2*^ *=22.82, p<0.001*). The number of needle partners among IDUs in the past 30 days did not differ between genders.

**Table 2:** Comparison of injecting drug-use behaviours among IDUs in the past 30 days.

	**Total (n=1724, %)**	**Male IDU(n=1528, %)**	**Female IDU(n=196, %)**	**Chi-2 Test *P* (*χ*^*2*^)**
**Shared syringes in the last 30 days**				
*Yes*	241 (14.0)	214 (14.0)	27 (13.8)	0.930 (0.008)
*No*	1483 (86.0)	1314 (86.0)	169 (86.2)	
**Injecting frequency in the last 30 days**				
*1-50*	386 (22.4)	343 (22.4)	43 (21.9)	0.001 (16.15)
*51-100*	803 (46.6)	733 (48.0)	70 (35.7)	
*101-150*	414 (24.0)	352 (23.0)	62 (31.6)	
*>150*	106 (6.7)	95 (6.2)	21 (10.8)	
*missing*	5 (0.3)	5 (0.3)	0 (0.0)	
*Average* (Mean±SD)	86.6±55.5	*85.6±54.4*	*94.8±62.6*	
**Sharing frequency in the last 30 days**				
*1-50*	174 (72.2)	160 (74.8)	14 (51.9)	0.004 (13.24)
*51-100*	36 (14.9)	31 (14.5)	5 (18.5)	
*101-150*	16 (6.6)	10 (4.7)	6 (22.2)	
*>150*	7 (2.9)	6 (2.8)	1 (3.7)	
*missing*	8 (3.3)	7 (3.3)	1 (3.7)	
*Average* (Mean±SD)	35.4±60.4	32.5±60.2	58.3±57.9	
**Number of needle partners in the last 30 days**				
0	68 (28.2)	59 (27.6)	9 (33.3)	0.118 (5.87)
*1*	107 (44.4)	92 (43.0)	15 (55.6)	
*2-5*	62 (35.7)	60 (28.0)	2 (7.4)	
*>5*	4 (1.6)	3 (1.4)	1 (3.7)	
*Average* (Mean±SD)	1.4±2.6	1.4±2.6	1.3±2.5	

* those inject in the past 30 days

In general, 64.9% of IDUs had had sex in the past three months, significantly lower than that of non-IDUs (71.8%, *G* ^*2*^ *=8.20, p=0.004*). However, among those who had had sex, the percentage of those who more than one sex partner was comparable between IDUs (6.8%) and non-IDU (4.2%), but the percentage of condom use was significantly lower in IDUs (20.5%) than non-IDUs (27.3%, *G* ^*2*^ *=4.74, p=0.030*). Female drug users were more likely to have had sex in the past three months than males (71.6% female versus 65.6% male, *G* ^*2*^ *=30.22, p<0.001*), but the number of sexual partners and condom usage did not differ.

### Disease burden and associated factors

Infectious disease burdens are high among MMT entrants; the prevalence of HIV, HCV and TB were 6.3%, 78.7% and 4.4% respectively. In addition, 5.6%, 0.5%, 3.8% and 0.3% of the MMT entrants were co-infected with HIV/HCV, HIV/TB, HCV/TB and HIV/HCV/TB respectively.

**Figure 1:**
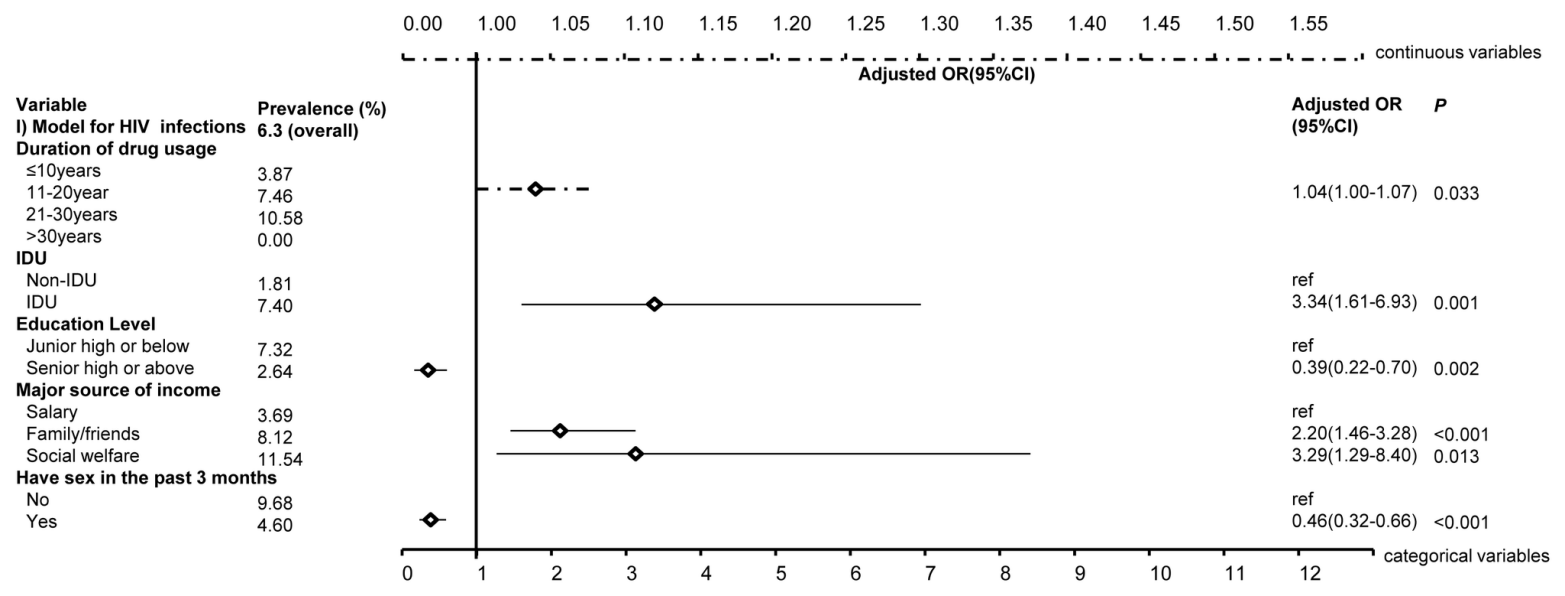
Significant associated factors for HIV infections among MMT entrants by multivariate regression analysis. The figure demonstrates the adjusted odds ratios (together with 95% confidence intervals) of factors that are significant associated with HIV infections among MMT entrants. The top dashed line denotes the scale for the continuous variables, whereas the bottom solid line denotes that for categorical variables.

**Figure 2:**
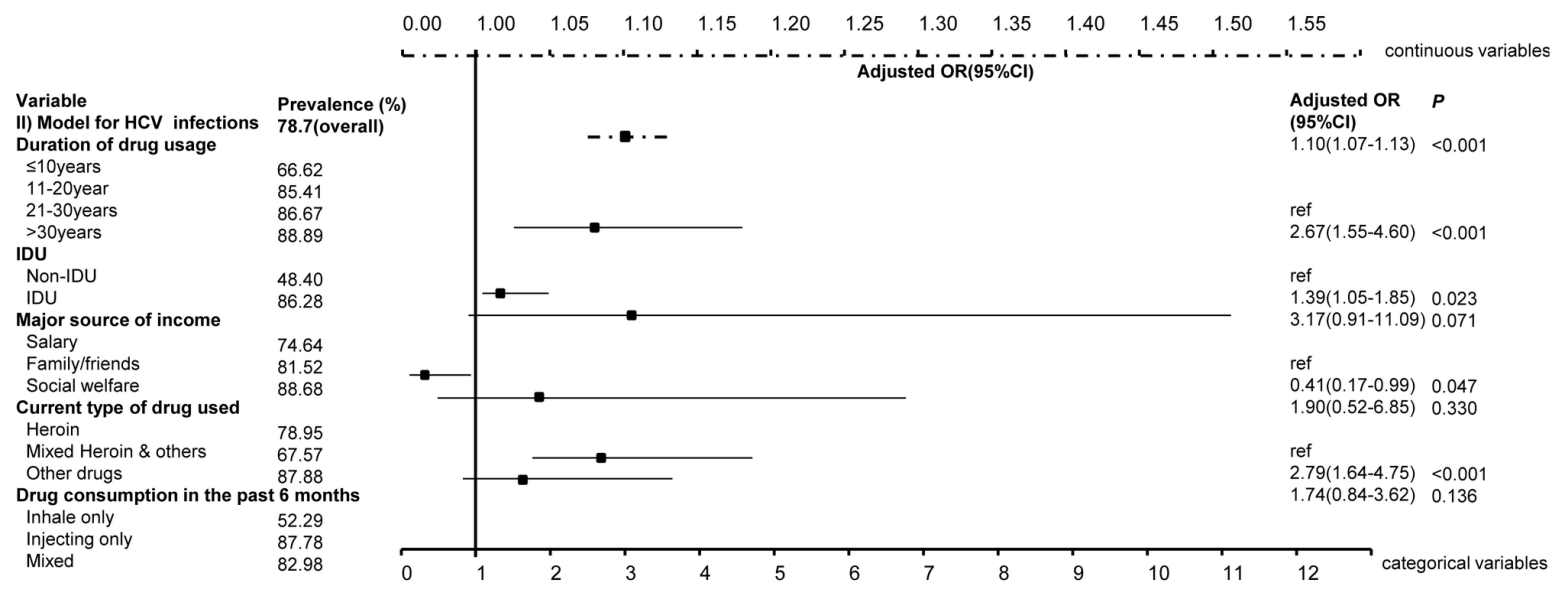
Significant associated factors for HCV infections among MMT entrants by multivariate regression analysis. The figure demonstrates the adjusted odds ratios (together with 95% confidence intervals) of factors that are significant associated with HCV infections among MMT entrants. The top dashed line denotes the scale for the continuous variables, whereas the bottom solid line denotes that for categorical variables.

**Figure 3:**
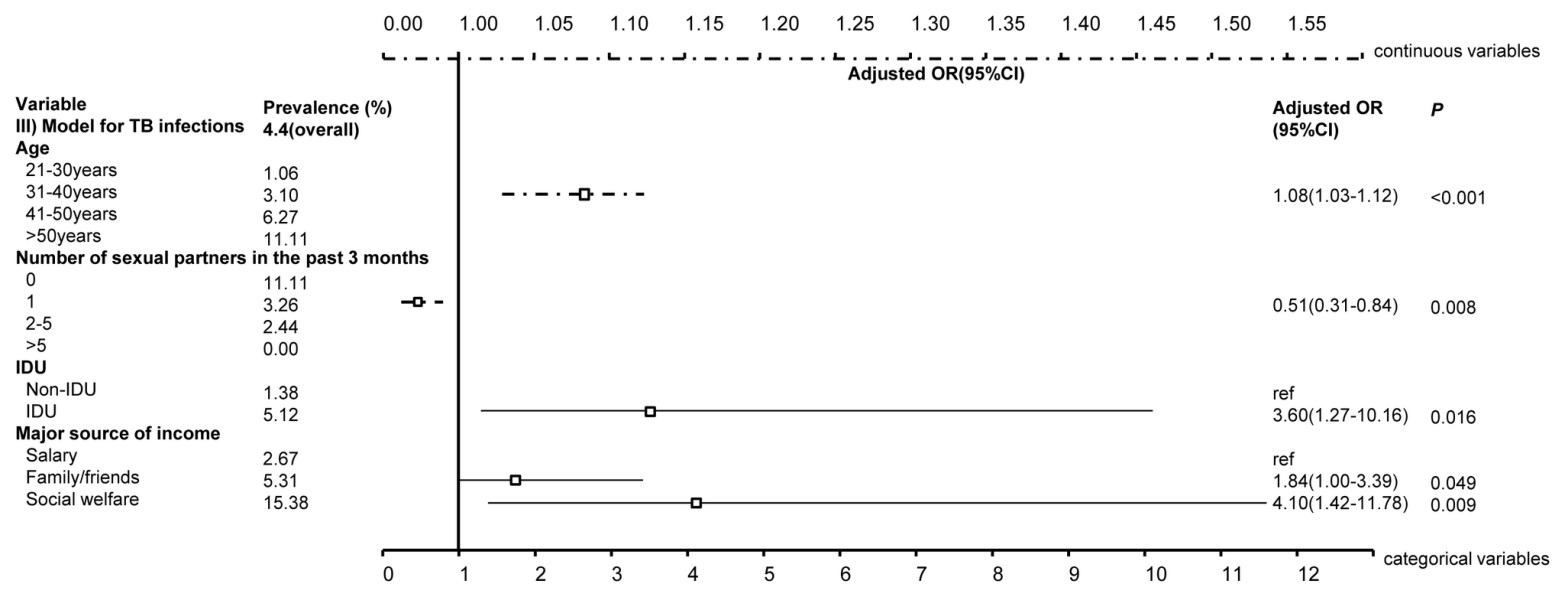
Significant associated factors for TB infections among MMT entrants by multivariate regression analysis. The figure demonstrates the adjusted odds ratios (together with 95% confidence intervals) of factors that are significant associated with TB infections among MMT entrants. The top dashed line denotes the scale for the continuous variables, whereas the bottom solid line denotes that for categorical variables.

Multivariate regression analysis indicated that being an IDU (adjusted OR= 3.34, 95% CI: 1.61-6.93, Figure 1) and financial dependence on family (aOR= 2.20, 1.46-3.28) and social welfare (aOR = 3.29, 1.29-8.40) were associated with higher odds of HIV infection, whereas a higher education level and having had sex in the past three months were associated with lower risk of HIV infection (aOR =0.39, 0.22-0.70 and aOR =0.46, 0.32-0.66, respectively). Every extra year of drug use significantly increased the risk of HIV infection by 4% (0-7%). Similarly, IDUs (aOR =2.67, 1.55-4.60, Figure 2), individuals who financially relied on family (aOR =1.39, 1.05-1.85) and had injecting as their sole means of drug consumption in the past six months (aOR =2.79, 1.64-4.75) have higher odds of HCV infection. The risk of HCV infection increased by 10% (7-13%) with every additional year of drug use, whereas mixed drug use contributed to a lower risk of HCV infection (aOR =0.41, 0.17-0.99). Notably, 89% of the HIV-infected entrants were also co-infected with HCV, indicating the urgency in providing antiretroviral drugs and HCV medications to individuals living with the co-infection. Consistently, higher TB infection is associated with individuals’ injecting profile (aOR =1.08, 1.03-1.12, Figure 3) and financial dependence on social welfare (aOR =4.10, 1.42-11.78); the odds of TB infection increased by 8% (3-12%) with any extra year of age. Notably, gender is not a significant contributing factor to HIV, HCV and TB infections when the confounding effects of drug-use duration were included (Table S1).

## Discussion

This study recruited 2296 MMT program entrants from four MMT clinics in Guangdong province. The sample represents a drug user population in their late 30s, most of whom are injectors, married or divorced, unemployed and financially dependent on family and social welfare. All infection risks of HIV, HCV and TB are consistently associated with increasing long-term drug use, injecting drugs, financial dependence and reduced sexual activities. Female IDUs demonstrate significantly higher injecting and sharing frequencies and lower rates of condom use at the last sexual intercourse than male IDUs and non-injecting drug users.

This study reports high HIV (6.3%), HCV (78.7%) and co-infection (5.6%) prevalence levels among MMT entrants. By contrast, based on a comprehensive meta-analysis of thirteen studies among MMT entrants in Guangdong, Zhuang et al. reported corresponding prevalence levels to be 3.6% (1.6-8.3%), 63.7% (25.2-90.1%) and 1.9% (0.9-3.8%) [9]. Our estimates tend to be higher but remain in the reported uncertainty bounds. This probably reflects the selection bias of the urban clinics located in neighbourhoods with large population of IDUs and people living with HIV in our study. In particular, the exceptionally high proportion (~90%) of HCV co-infection among HIV-positive entrants is also consistent with a previous study [6], highlighting the importance of integrated treatment for HCV among MMT participants, especially for those co-infected with HIV. Notably, this study reveals a relatively high TB prevalence (4.4%) but its co-infection with HIV is low (0.5%). HIV-induced immunosuppression is the most common reason for a high TB infection rate among drug users [31], but it may not be the case in our study. Little is known about TB transmission among drug users in China, but illicit drug use is punishable by law and incarceration of drug users in compulsory detoxification centres and labour camps is common. Accumulated evidence indicates that these confined settings substantially add to the elevated risk of TB transmission among drug users [15,32].

Injecting drug use remains as the major risk for HIV, HCV and TB infections. The significantly higher HIV and HCV prevalence levels among IDUs in our findings is consistent with previous reports [21]. The higher infection risk is a result of greater risk behaviours among IDUs. IDUs consume drugs more frequently than non-injectors. The finding of an average 3.3-3.7 daily injections is comparable to other Asian countries with IDU-driven HIV epidemics [33,34], but is considerably higher than in developing countries settings [35-37]. Among injectors, 14% shared syringes in an average of 35 episodes in the past 30 days, highlighting the urgency of the provision of clean needle and syringes during the course of MMT. Further, IDUs have a substantially longer history of drug use, identified as a contributing factor of HIV and HCV infections both in this study and previous reports [38-40]. Most drug users start with inhaling, but as drug dosage increases they often switch to injecting drug use after several years of addiction [41-44]. High drug prices and continuing and frequent drug consumption quickly deplete any personal savings and revenues, and drug users, especially IDUs, become more financially dependent on family support and social security. The employment rate is significantly lower among our injecting participants, probably as a result of their physical weakness, inability to fulfil job duties due to addiction and criminal records of robbery and theft [36,45]. IDUs also report significantly more suppressed sexual drive than non-IDUs. Erectile and other sexual dysfunctions are commonly found among MMT participants with intensive drug use [46,47]. This is consistent with the study finding of an inverse association between risk of HIV infection and having had sex in the past three months.

Female drug users represent a minor (13%) but more vulnerable sub-population of drug users. HIV and behavioural studies among female drug users have been documented extensively in the international settings [48-52]. However, it has only come under the spotlight in China in recent years as overlapping drug use and female sex work has been reported [53,54]. Lau et al have published a series of studies specifically on female IDUs, systematically demonstrating high levels of syringe-sharing, multiple concurrent sexual partnerships and unprotected sexual intercourse in this sub-population [55,56]. Adding to these findings, the demographic and behavioural analysis in this study indicates that female drug users are more disadvantaged in their social status and risk-taking in their drug use behaviours than their male counterparts. Being less financially independent means females have greater difficulties abstaining from drug use if their concordant male partners continue to support their drug consumption. Interestingly, our result of more prevalent injecting and sharing behaviours among female drug users contradicts the established findings [57-61] but aligns with similar studies in correctional and confined settings [62-64]. In these settings, female detainees reportedly have poorer mental health and experience greater physical and sexual abuse [65,66], and both of these are contributing factors in drug use by females [66,67]. Incarceration of drug users for compulsory detoxification is common in China [19,68,69]; whether this has contributed to the poor mental health and hence more risk-taking drug use among female drug users remain necessary for future exploration. Despite greater risk behaviours, disease burdens in females are not substantially higher than in males. The greater risk behaviours are probably counteracted by the significantly shorter drug use history and hence less cumulative risks for infections among females.

Several limitations in this study should be noted. First, 5-10% of the blood sample test results were missing from the database. However, this only accounts for a small proportion of the total sample and does not affect the study conclusions. Second, this prospective study identified the associated factors of infections, but is not an evidence of causalities. Third, self-reported behaviours of drug users may suffer from recall and social desirability biases. Fourth, chest X-ray diagnosis of TB has low sensitivity and specificity. The estimated prevalence of TB in the participants needs to be interpreted with caution. Integration of MMT clinic and hospital information systems may provide more accurate confirmation of TB status. Finally, our study was conducted in four selected MMT clinics in high HIV transmission areas of urban Guangdong; extrapolation of the study implications might be limited.

Achieving the full potential benefits of the MMT program requires a comprehensive integration with other peripheral interventions. As drug injecting remains the major risk of HIV and HCV infections, other harm reduction strategies such as needle and syringe exchange programs should be made accessible for MMT participants, especially female injectors, in parallel to the current treatment programs. Scale-up of referral services, provision of HCV treatment and treatment of TB according to the WHO’s ‘Stop TB Strategy’ guidelines [70] are necessary to prevent further transmission of infections among MMT program participants in China.

## Supporting Information

Table S1 **Regression analysis of associated factors for HIV, HCV and TB infections among MMT entrants.**
(DOCX)
