# Supplementary material for: High Prevalence of HIV, HCV and Tuberculosis and Associated Risk Behaviours among New Entrants of Methadone Maintenance Treatment Clinics in Guangdong Province, China
Source: PLoS One. 2013 Oct 8;8(10):e76931. doi: 10.1371/journal.pone.0076931 (PMC3792874; doi:10.1371/journal.pone.0076931)
Supplement: Table S1 — Regression analysis of associated factors for HIV, HCV and TB infections among MMT entrants. (DOCX) [file pone.0076931.s001.docx]

**Supplementary Material**

**Table S1: Regression analysis of associated factors for HIV, HCV and TB infections among MMT entrants.**

|  | **HIV** | | | | **HCV** | | | | | **TB** | | | | |
| --- | --- | --- | --- | --- | --- | --- | --- | --- | --- | --- | --- | --- | --- | --- |
|  | **Counts (n)** | **Prev (%)** | **Bivariate Reg. OR (95% CI)** | **Multivariate Reg. OR (95% CI)** | **Counts(n)** | **Prev (%)** | **Bivariate Reg. OR (95% CI)** | **Multivariate Reg. OR (95% CI)** | | **Counts(n)** | **Prev (%)** | | **Bivariate Reg. OR (95% CI)** | **Multivariate Reg. OR (95% CI)** |
| ***Demographic Characteristics*** | | | | | | | | | | | | | | |
| **IDU** | | | | | | | | | | | | | | |
| Non-IDU | 443 | 1.81 | ref | ref | 438 | 48.40 | ref | ref | | 434 | | 1.38 | ref | ref |
| IDU | 1744 | 7.40 | 4.34 (2.11-8.94)** | 3.34 (1.61-6.93)** | 1742 | 86.28 | 6.70 (5.32-8.45)** | 2.67 (1.55-4.60)** | | 1700 | | 5.12 | 3.85 (1.67-8.86)** | 3.60 (1.27-10.16)* |
| **Gender** | | | | | | | | | | | | | | |
| female | 274 | 5.47 | ref |  | 273 | 73.63 | ref |  | | 269 | | 0.74 | ref |  |
| male | 1913 | 6.38 | 1.18 (0.68-2.04) |  | 1907 | 79.39 | 1.38 (1.03-1.85)* |  | | 1865 | | 4.88 | 6.85 (1.68-27.96)** |  |
| **Age** | | | | | | | | | | | | | | |
| 21-30 | 201 | 3.98 |  |  | 198 | 68.69 |  |  | | 189 | | 1.06 |  |  |
| 31-40 | 1177 | 6.46 | 1.01 (0.98-1.04) |  | 1169 | 77.59 | 1.04 (1.02-1.05)** |  | | 1128 | | 3.10 | 1.09 (1.06-1.12)** | 1.08 (1.03-1.12)** |
| 41-50 | 714 | 6.86 |  |  | 716 | 82.26 |  |  | | 718 | | 6.27 |  |  |
| >50 | 95 | 4.21 |  |  | 97 | 85.57 |  |  | | 99 | | 11.11 |  |  |
| **Marital status** | | | | | | | | | | | | | | |
| Single | 1014 | 7.59 | ref |  | 1015 | 81.77 | ref |  | | 997 | | 5.22 | ref |  |
| Married | 940 | 5.00 | 0.64 (0.44-0.93)* |  | 935 | 74.65 | 0.66 (0.53-0.82)** |  | | 907 | | 3.31 | 0.62 (0.39-0.98)* |  |
| Divorced/widowed | 233 | 5.58 | 0.72 (0.39-1.32) |  | 230 | 81.30 | 0.97 (0.67-1.40) |  | | 230 | | 4.78 | 0.91 (0.47-1.78) |  |
| **Education level** | | | | | | | | | | | | | | |
| Junior high or below | 1694 | 7.32 | ref | ref | 1696 | 81.13 | ref |  | | 1657 | | 4.35 | ref |  |
| Senior high or above | 493 | 2.64 | 0.34 (0.19-0.61)** | 0.39 (0.22-0.70)* | 484 | 70.04 | 0.54 (0.43-0.68)** |  | | 477 | | 4.40 | 1.01 (0.62-1.67) |  |
| **Major source of income** | | | | | | | | | | | | | | |
| Salary | 975 | 3.69 | ref | ref | 970 | 74.64 | ref | ref | | 938 | | 2.67 | ref | ref |
| Family/friends | 1145 | 8.12 | 2.31 (1.55-3.42)** | 2.20 (1.46-3.28)** | 1142 | 81.52 | 1.50 (1.22-1.85)** | 1.39 (1.05-1.85)* | | 1129 | | 5.31 | 2.05 (1.27-3.30)** | 1.84 (1.00-3.39)* |
| Social welfare | 52 | 11.54 | 3.40 (1.37-8.48)** | 3.29 (1.29-8.40)* | 53 | 88.68 | 2.66 (1.12-6.30)* | 3.17(0.91-11.09) | | 52 | | 15.38 | 6.64 (2.83-15.56)** | 4.10 (1.42-11.78)* |
| ***Risk drug-use behaviours*** | | | | | | | | | | | | | | |
| **Current type of drug used** | | | | | | | | | | | | | | |
| Heroin | 2080 | 6.25 | ref |  | 2076 | 78.95 | ref | ref | | 2031 | | 4.43 | ref |  |
| Mixed Heroin & others | 38 | 5.26 | 0.83 (0.20-3.50) |  | 37 | 67.57 | 0.56 (0.28-1.11) | 0.41(0.17-0.99)* | | 35 | | 2.86 | 0.63 (0.09-4.69) |  |
| Other drugs | 33 | 9.09 | 1.50 (0.45-4.98) |  | 33 | 87.88 | 1.93 (0.68-5.53) | 1.90(0.52-6.85) | | 33 | | 3.03 | 0.67 (0.09-4.99) |  |
| **Drug consumption in the past 6 months** | | | | | | | | | | | | | | |
| Inhale only | 550 | 2.18 | ref |  | 545 | 52.29 | ref | ref | | 536 | | 1.87 | ref |  |
| Injecting only | 1484 | 8.02 | 3.91 (2.14-7.14)** |  | 1481 | 87.78 | 6.55 (5.21-8.24)** | 2.79 (1.64-4.75)** | | 1453 | | 5.09 | 2.82 (1.45-5.50)** |  |
| Mixed | 140 | 4.29 | 2.01 (0.74-5.45) |  | 141 | 82.98 | 4.45 (2.78-7.12)** | 1.74 (0.84-3.62) | | 132 | | 5.30 | 2.95 (1.10-7.89)* |  |
| **Frequency of daily drug usage in the last 30 days** | | | | | | | | | | | | | | |
| *≤1 times / day* | 159 | 3.77 |  |  | 157 | 71.97 |  |  | | 152 | | 1.97 |  |  |
| *1-2 times / day* | 520 | 5.58 | 1.36 (1.06-1.74)** |  | 525 | 80.19 | 1.00 (0.98-1.01) |  | | 514 | | 4.28 | 0.98 (0.93-1.03) |  |
| *3-5 times / day* | 1315 | 6.54 |  |  | 1308 | 79.20 |  |  | | 1276 | | 4.94 |  |  |
| *>5times /day* | 161 | 9.94 |  |  | 159 | 77.99 |  |  | | 162 | | 3.09 |  |  |
| **Injecting drugs in the last 30 days** | | | | | | | | | | | | | | |
| No | 543 | 2.58 | ref |  | 537 | 54.56 | ref |  | | 530 | | 1.51 | ref |  |
| Yes | 1641 | 7.50 | 3.06 (1.75-5.37)** |  | 1640 | 86.65 | 5.40 (4.33-6.74)** |  | | 1601 | | 5.31 | 3.66 (1.76-7.60)** |  |
| **Duration of drug usage** | | | | | | | | | | | | | | |
| *≤10 year* | 801 | 3.87 |  |  | 791 | 66.62 |  |  | | 769 | | 2.86 |  |  |
| *11-20 year* | 1273 | 7.46 | 1.05 (1.02-1.08)** | 1.04 (1.00-1.07)* | 1275 | 85.41 | 1.13 (1.10-1.15)** | 1.10 (1.07-1.13)** | | 1251 | | 5.04 | 1.05 (1.01-1.09)** |  |
| *21-30 years* | 104 | 10.58 |  |  | 105 | 86.67 |  |  | | 106 | | 7.55 |  |  |
| >*30 years* | 9 | 0.00 |  |  | 9 | 88.89 |  |  | | 8 | | 0.00 |  |  |
| ***Risk sexual behaviours*** | | | | | | | | | | | | | | |
| **Have sex in the past 3 months** | | | | | | | | | | | | | | |
| No | 713 | 9.68 | ref | ref | 714 | 81.79 | ref |  | | 710 | | 4.37 | ref |  |
| Yes | 1456 | 4.60 | 0.45 (0.32-0.64)** | 0.46(0.32-0.66)** | 1446 | 77.25 | 0.76 (0.60-0.95)* |  | | 1406 | | 4.41 | 1.01 (0.65-1.57) |  |
| **Number of sexual partners in the past 3 moths** | | | | | | | | | | | | | | |
| 0 | 229 | 7.86 |  |  | 227 | 85.46 |  |  | | 225 | | 11.11 |  |  |
| 1 | 1081 | 4.07 | 0.95 (0.76-1.19) |  | 1073 | 75.58 | 1.02 (0.97-1.07) |  | | 1043 | | 3.26 | 0.35 (0.21-0.57)** | 0.51 (0.31-0.84)* |
| 2-5 | 131 | 3.05 |  |  | 131 | 77.10 |  |  | | 123 | | 2.44 |  |  |
| >6 | 12 | 8.33 |  |  | 12 | 83.33 |  |  | | 3 | | 0.00 |  |  |
| **Condom Usage in the last sex act** | | | | | | | | | | | | | | |
| No | 976 | 4.10 | ref |  | 971 | 78.58 | ref | |  | 938 | | 5.22 | ref |  |
| Yes | 478 | 5.86 | 1.46 (0.89-2.39) |  | 474 | 74.68 | 0.80 (0.62-1.04) | |  | 467 | | 3.21 | 0.6 (0.33-1.09) |  |
| **Have sex under the influence of drug in the past 3 months** | | | | | | | | | | | | | | |
| No | 2130 | 6.15 | ref |  | 2123 | 78.90 | ref | |  | 2081 | | 4.37 | ref |  |
| Yes | 31 | 12.90 | 2.26 (0.78-6.56) |  | 31 | 70.97 | 0.65 (0.30-1.43) | |  | 27 | | 7.41 | 1.75 (0.41-7.50) |  |

*P<0.05,**P≤0.001
